# Curious Coexistence of Perivascula Epitheloid Cell Tumour (PEComa) Systemic Sclerosis-Associated Interstitial Lung Disease and Biopsy-Proven Lymphangioleiomyomatosis in a Middle-Aged Female: A Case Report

**DOI:** 10.7759/cureus.95884

**Published:** 2025-11-01

**Authors:** Said Isse, Irfan Shafiq, Haytham Dimashkieh, Asif Quadri, Saniya Khan

**Affiliations:** 1 Respiratory Medicine, Cleveland Clinic, Abu Dhabi, ARE; 2 Respiratory and Allergy Medicine, Cleveland Clinic, Abu Dhabi, ARE; 3 Pathology and Laboratory Medicine, Cleveland Clinic, Abu Dhabi, ARE

**Keywords:** case report, ild, lymphangioleiomyomatosis (lam), mycophenolate, pecomas, sirolimus, systemic sclerosis interstitial lung disease (ssc-ild)

## Abstract

We describe a rare case of an unusual coexistence of systemic sclerosis-associated interstitial lung disease (SSc-ILD), biopsy-proven lymphangioleiomyomatosis (LAM), and perivascular epithelioid cell tumor (PEComa) in a 41-year-old woman. Initial presentation with retroperitoneal angiomyolipoma preceded the later development of diffuse cystic lung disease and features of systemic sclerosis. Histopathology confirmed both LAM and interstitial pneumonitis. The patient improved on mycophenolate for SSc-ILD, with sirolimus planned for LAM. This unique case highlights the diagnostic challenges and the importance of multidisciplinary management in overlapping rare pulmonary and systemic diseases.

## Introduction

Lymphangioleiomyomatosis (LAM) is an uncommon, multisystem neoplastic disease characterised by cystic lung disease, leading to progressive dyspnoea, recurrent pneumothorax, and chylous effusions, as well as abdominal tumours such as angiomyolipomas and lymphangioleiomyomas. The pathological hallmark is typified by smooth muscle proliferation affecting the lungs, lymphatics, and kidneys. LAM typically affects women of childbearing age and may be sporadic or associated with tuberous sclerosis complex (TSC) [1].

Perivascular epithelioid cell tumors (PEComas) are a family of rare mesenchymal neoplasms composed of distinctive perivascular epithelioid cells showing both melanocytic and smooth muscle differentiation. They encompass angiomyolipomas, lymphangioleiomyomas, and related tumors, many of which share pathogenic activation of the mammalian target of rapamycin (mTOR) signaling pathway, particularly in association with tuberous sclerosis complex (TSC) [1].

The diagnosis of LAM requires a compatible clinical history, characteristic high-resolution chest CT (HRCT) changes, along with at least one of the following: presence of tuberous sclerosis complex (TSC), renal angiomyolipoma, elevated serum vascular endothelial growth factor-D (VEGF-D) ≥800 pg/mL, chylous effusion, lymphangioleiomyomas, demonstration of LAM cells or clusters in effusions or lymph nodes, or histopathological confirmation from lung or extrapulmonary biopsy [2].

Systemic sclerosis (SSc) is a chronic autoimmune connective tissue disease characterised by widespread vascular dysfunction, immune activation, and progressive fibrosis of the skin and internal organs [3].

Systemic sclerosis-associated interstitial lung disease (SSc-ILD) is a common and potentially severe complication of systemic sclerosis, with HRCT detecting interstitial lung disease in up to 80% of patients. However, only 25-30% develop a clinically significant disease that may progress to respiratory failure or death. SSc-ILD commonly presents with dyspnoea, cough, and a non-specific interstitial pneumonia pattern on HRCT [4].

While both LAM and SSc can cause significant pulmonary morbidity, their concurrence in a single patient is extremely rare and poses unique diagnostic and therapeutic challenges. This case report describes a patient presenting with both biopsy-proven LAM, SSc-ILD, and PEComas, providing insights into the clinical course and management of this unusual combination.

## Case presentation

**Figure 1:**
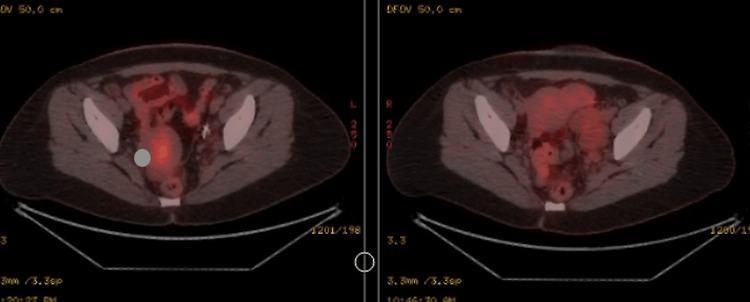
PET/CT imaging of retroperitoneal angiomyolipoma (PEComa) Axial fused PET/CT images demonstrate a well-circumscribed retroperitoneal mass (left panel, oval shape) located adjacent to the left adnexa, showing mild FDG uptake. The right panel shows interval postoperative imaging with stable postoperative changes and no new hypermetabolic lesions. PEComa - perivascular epithelioid cell tumor

A 41-year-old female without any significant past medical or smoking history presented in January 2020 with menorrhagia and abdominal pain, leading to the discovery of a left ovarian mass on MRI. In March 2020, she underwent surgery for a retroperitoneal tumour excision. A residual tumour measuring approximately 1 cm was left behind due to its vascular involvement. Histopathology confirmed the mass to be a retroperitoneal angiomyolipoma. Subsequent CT and MRI scans showed no progression in the size of the residual pelvic angiomyolipoma. However, she later developed pelvic and para-aortic lymph nodes, which showed no FDG uptake on the PET scan (Figure 1).

**Figure 2:**
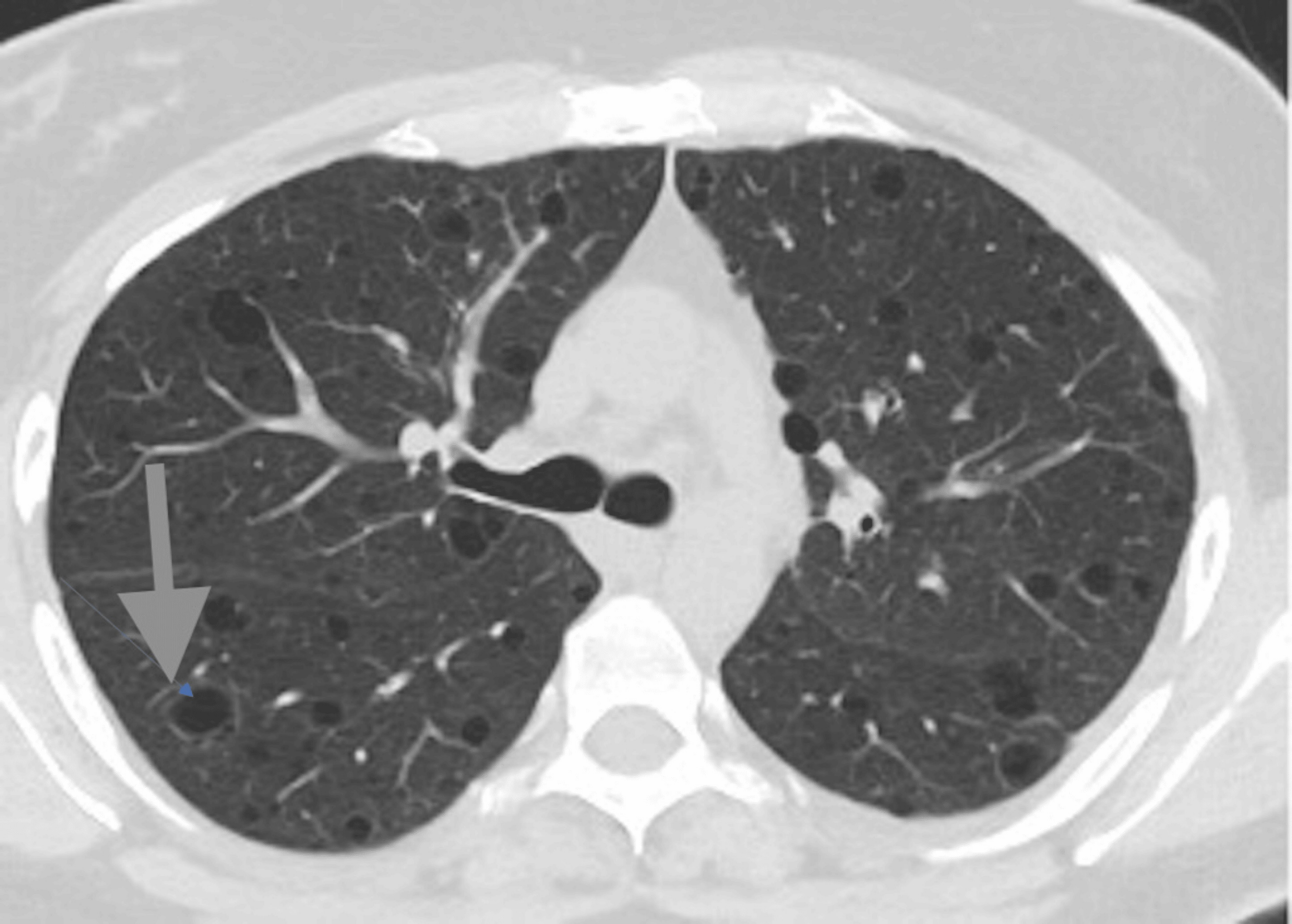
High-resolution computed tomography of the chest HRCT demonstrates innumerable thin-walled cysts distributed bilaterally (arrowheads), characteristic of lymphangioleiomyomatosis. No consolidation or nodules are observed. Image obtained using standard HRCT protocol (1 mm collimation) HRCT - high-resolution computed tomography

In early October 2023, the patient experienced chest pain and shortness of breath. High-resolution computed tomography (HRCT) of the chest (Figure 2) revealed widespread, thin-walled cystic lesions bilaterally throughout both lung fields, raising concern for LAM. These lesions were retrospectively identified on her 2020 PET CT scan, where they had been mischaracterised as emphysematous changes. 

**Figure 3:**
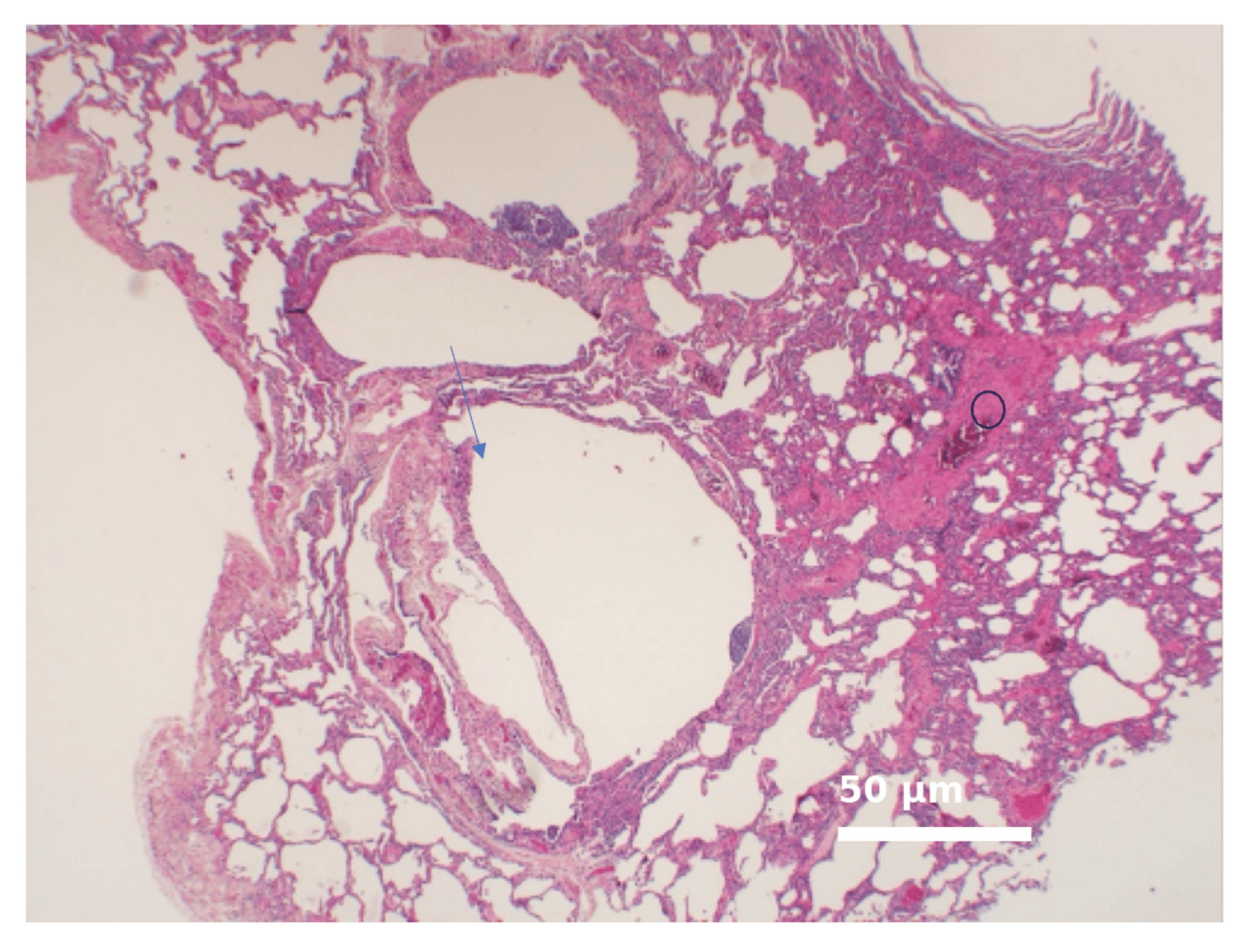
Histopathology of lung biopsy Representative photomicrograph showing cystic spaces lined by abnormal smooth muscle cells typical of LAM (arrowheads), alongside chronic interstitial pneumonitis with lymphoplasmacytic infiltrates and focal bronchiolitis (open circle). Hematoxylin and Eosin stain; magnification ×200. Scale bar = 50 μm. LAM - lymphangioleiomyomatosis

Further evaluation revealed symptoms consistent with systemic sclerosis, including a new-onset rash, dry skin on the forearms, cold fingers (Raynaud's phenomenon), facial skin tightness, and reduced mouth opening. Skin involvement was quantified using the Modified Rodnan Skin Score (mRSS), which was 12 (out of 51) at baseline, indicating mild-to-moderate limited cutaneous systemic sclerosis, and improved to six after 12 months of mycophenolate mofetil therapy [5]. She also reported chronic abdominal pain, early satiety, chronic nausea, malaise, fatigue, and weakness. Her routine laboratory findings and clinic visit vitals are shown in Table 1. Subsequent transbronchial biopsy (Figure 3) showed changes consistent with LAM.

**Table 1:** Laboratory results and vitals Routine laboratory examinations and vitals at presentation. Reference ranges provided in accordance with institutional standards. WBC - white blood cell; ALT - alanine aminotransferase; AST - aspartate aminotransferase; ANA - antinuclear antibody; ENA - extractable nuclear antigens; VEGF-D - vascular endothelial growth factor-D; BP - blood pressure; HR - heart rate

Test	Result	Reference range	Units
Hemoglobin	13.4	12-15	g/dL
WBC	6.2	4-10	×10⁹/L
Platelets	240	150-400	×10⁹/L
Creatinine	0.8	0.6-1.2	mg/dL
ALT	21	<40	U/L
AST	19	<40	U/L
ANA	Negative	Negative	-
ENA	Negative	Negative	-
Scl-70 antibody	>8.0	0.0-0.9	AI
VEGF-D	24	0-115	pg/mL
O₂ saturation	96	>95	%
BP	118/74	90-140/60-90	mmHg
HR	82	60-100	bpm

Treatment and clinical course

The patient was started on mycophenolate mofetil 1 g twice daily in November 2023 for systemic sclerosis-associated interstitial lung disease. She tolerated therapy without gastrointestinal or hematological complications. Over the subsequent 12 months, her skin thickening, Raynaud's phenomenon, and fatigue improved significantly. Serial pulmonary function tests (Table 2) demonstrated gradual stabilization and mild improvement in forced vital capacity (FVC) and diffusing capacity for carbon monoxide (DLCO).

**Table 2:** Pulmonary function tests over time Pulmonary function test results demonstrating obstructive ventilatory defect with reduced diffusion capacity, with partial improvement over time. Predicted values based on age, sex, and height. FVC - forced vital capacity; FEV1 - forced expiratory volume in 1 second; DLCO - diffusing capacity for carbon monoxide

Date	FVC ( %pred), L	FEV1 ( %pred), L	FEV1/FVC (%)	DLCO (%pred)
Nov 2023	1.82 (58%)	1.67 (66%)	113	40%
Jun 2024	2.05 (56%)	1.14 (38%)	56	65%
Oct 2024	2.23 (61%)	1.32 (44%)	59	70%

Although sirolimus is the standard therapy for lymphangioleiomyomatosis (LAM) and mycophenolate mofetil is first-line for systemic sclerosis-associated interstitial lung disease (SSc-ILD), there are currently no prospective trials or guideline-endorsed data evaluating their combined or sequential use in patients with both conditions. Sirolimus initiation was deferred to allow evaluation of mycophenolate efficacy and tolerability; initiation is planned within 6-12 months, targeting stabilization of LAM-related cystic lung disease and prevention of further angiomyolipoma growth.

On follow-up through August 2025, the patient remains clinically stable, reports improved exercise tolerance, and has no recurrence of abdominal pain or new angiomyolipomas. Close multidisciplinary surveillance continues.

## Discussion

This case illustrates a rare concomitant occurrence of LAM and SSc-ILD, presenting a complex diagnostic and therapeutic challenge. Our literature search did not yield case reports detailing the co-occurrence of LAM and SSc, highlighting the unique nature of this presentation. The patient's initial presentation with a retroperitoneal angiomyolipoma, a tumour often associated with TSC and LAM [1, 2], provided an early clue, though the lung cysts were initially misinterpreted. The subsequent development of widespread lung cysts and classic SSc symptoms necessitated a thorough investigation, ultimately leading to the biopsy-proven diagnosis of both conditions. The low VEGF-D level was discussed as a known limitation in LAM diagnosis, emphasizing that VEGF-D <800 pg/mL does not exclude LAM when histopathologic confirmation is present.

LAM and SSc-ILD are distinct diseases with different pathophysiologies. LAM is characterised by the destructive proliferation of LAM cells, leading to cystic changes, while SSc-ILD is a fibrotic process driven by autoimmune mechanisms [1, 3]. The presence of chronic interstitial pneumonitis with lymphoplasmacytic infiltrates and focal bronchiolitis in our patient's lung biopsy, alongside the classic LAM cysts, suggests an overlap or a separate inflammatory process. The pathologist's comment regarding the atypical inflammation pattern for LAM and the consideration of chronic silent aspiration in the setting of scleroderma is particularly insightful [4]. Gastrointestinal involvement is common in SSc, and oesophageal dysmotility can lead to aspiration, potentially contributing to the interstitial changes observed [6].

The management of this patient requires a delicate balance. Mycophenolate is a standard immunosuppressant used for SSc-ILD to slow disease progression and improve lung function [7]. At the same time, sirolimus (rapamycin) is the only FDA-approved therapy for LAM, shown to stabilise lung function and reduce chylous effusions and angiomyolipoma size [8]. The choice to delay sirolimus initiation until after 6-12 months of mycophenolate therapy reflects a careful approach, aiming to optimise the SSc component initially while assessing the tolerability of mycophenolate before adding another immunosuppressive agent. The patient's overall improvement in both pulmonary function, skin, and constitutional symptoms on mycophenolate is encouraging, suggesting a positive response to SSc treatment [9].

The presence of PEComa requires ongoing surveillance [10], as these tumors are also associated with TSC and LAM [11]. The multidisciplinary approach, involving pulmonology, rheumatology, gynecology, oncology, and pathology, was vital for accurate diagnosis and comprehensive management in this complex case.

## Conclusions

This case report describes an unusual example of systemic sclerosis with interstitial lung disease co-occurring with lymphangioleiomyomatosis. The patient's clinical course underlines the diagnostic challenges and the need for a high index of suspicion when atypical features or multiple system involvement are present. The successful management with mycophenolate for SSc and planned Sirolimus for LAM emphasises the importance of individualised, multidisciplinary care in such complex cases. Further research is needed to understand the potential pathogenic links or unique clinical implications of these rare co-morbidities.
